# Automatically extracting functionally equivalent proteins from SwissProt

**DOI:** 10.1186/1471-2105-9-418

**Published:** 2008-10-06

**Authors:** Lisa EM McMillan, Andrew CR Martin

**Affiliations:** 1 Research Department of Structural & Molecular Biology, University College London, Gower Street, London WC1E 6BT, UK

## Abstract

**Background:**

There is a frequent need to obtain sets of functionally equivalent homologous proteins (FEPs) from different species. While it is usually the case that orthology implies functional equivalence, this is not always true; therefore datasets of orthologous proteins are not appropriate. The information relevant to extracting FEPs is contained in databanks such as UniProtKB/Swiss-Prot and a manual analysis of these data allow FEPs to be extracted on a one-off basis. However there has been no resource allowing the easy, automatic extraction of groups of FEPs – for example, all instances of protein C.

We have developed FOSTA, an automatically generated database of FEPs annotated as having the same function in UniProtKB/Swiss-Prot which can be used for large-scale analysis. The method builds a candidate list of homologues and filters out functionally diverged proteins on the basis of functional annotations using a simple text mining approach.

**Results:**

Large scale evaluation of our FEP extraction method is difficult as there is no gold-standard dataset against which the method can be benchmarked. However, a manual analysis of five protein families confirmed a high level of performance. A more extensive comparison with two manually verified functional equivalence datasets also demonstrated very good performance.

**Conclusion:**

In summary, FOSTA provides an automated analysis of annotations in UniProtKB/Swiss-Prot to enable groups of proteins already annotated as functionally equivalent, to be extracted. Our results demonstrate that the vast majority of UniProtKB/Swiss-Prot functional annotations are of high quality, and that FOSTA can interpret annotations successfully. Where FOSTA is not successful, we are able to highlight inconsistencies in UniProtKB/Swiss-Prot annotation. Most of these would have presented equal difficulties for manual interpretation of annotations. We discuss limitations and possible future extensions to FOSTA, and recommend changes to the UniProtKB/Swiss-Prot format, which would facilitate text-mining of UniProtKB/Swiss-Prot.

## Background

It is often necessary to compare the 'same' gene or gene product (protein) in different species. By the 'same' protein, we mean an orthologue that performs an equivalent function or functions. Obtaining lists of functionally-equivalent proteins (FEPs) is fundamental for comparative and evolutionary genomics, and downstream proteomic studies [1]. The particular motivation for the current work was obtaining lists of FEPs to examine residue conservation scores and to aid in understanding the effects of mutations on protein function in the context of a large-scale automated analysis pipeline, SAAPdb [2]. Proteins that have diverged in function (either by gaining or losing functionality) will show differences at key functional residues. We therefore needed a reliable automatic method for extracting groups of FEPs from UniProtKB/Swiss-Prot.

Consider, for example, the HOX family of genes, which is a large family of transcription factor proteins containing the well characterised homeobox motif. These proteins are well conserved across species and are believed to be critical in embryogenesis, oncogenesis and differentiation processes such as haematopoiesis [3,4]. HOX proteins are representative of large protein families in that there are several paralogues within a species (thirteen in the case of the human HOX family [3]), and each paralogue can be involved in several distinct aspects of the same biological process. A sequence alignment of such evolutionarily related, but functionally different, proteins would contain significant noise, and obscure much of the genuine functional conservation between true FEPs.

While homology does not imply functional equivalence, it is also not possible to use functional data alone to identify FEPs. Proteins can converge on similar functions without being evolutionarily related. For example, subtilisin (EC 3.4.21.62) and trypsin (EC 3.4.21.4) have evolved separately in bacteria and vertebrates respectively; they differ significantly in protein sequence, structure and fold, yet the same three amino acids form the catalytic triad in both proteins [5]. Aligning such functionally similar, but evolutionarily unrelated, proteins is meaningless; we are interested in proteins which are both homologous and functionally equivalent.

Two entities are homologous if they have a common evolutionary origin. An *orthologous *relationship denotes that this common origin was a speciation event, whereas *paralogues *are related by a gene duplication [6]. Paralogues, having been derived via a mechanism for functional divergence, are likely to perform different functions [7]. While orthologues generally perform the same function, it is possible for the function to diverge, particularly when orthologues are evolutionarily distant [6]. For example, Shibata *et al*. [8] showed that although the general function of exportin-5 proteins (nuclear export of miRNAs and tRNAs) is conserved across different species, substrate specificity varies. Further, the *AGAMOUS *gene in Arabidopsis is involved in carpel and stamen development, but the two orthologues in maize have specialised: *ZAG1 *is highly expressed during carpel development, and *ZMM2 *is expressed during stamen development [9]. It is clear then that orthology need not imply strict functional equivalence, and it follows that sets of orthologues, defined by methods such as Inparanoid [10], C/KOG [11,12] and TOGA [13], are not appropriate as lists of FEPs. Further, these methods are computationally intensive and as such are often limited to small species sets.

The identification of true FEPs requires consideration of features such as functional assays, interaction networks, expression data and so forth. UniProtKB/Swiss-Prot is a carefully annotated databank of protein sequences that includes functional annotations. While many of these are transferred through orthology, where there is experimental evidence for function, it will be included. Thus, short of conclusive experimental studies, the most reliable way of identifying families of FEPs is first to identify families of homologues in UniProtKB/Swiss-Prot and then to examine the annotations to find a set of proteins that are annotated as performing the same function or functions. It is, of course, possible that annotations in UniProtKB/Swiss-Prot will be incorrect, but as UniProtKB/Swiss-Prot is updated on a regular basis, it is expected that these annotations will represent the most up-to-date state of our knowledge of protein function, and errors in annotations will be corrected with future releases.

While it is perfectly possible to perform this analysis on an individual basis by searching UniProtKB/Swiss-Prot for homologues and comparing the annotations manually, there is a pressing need for an automatically updated resource that simply lists families of FEPs in UniProtKB/Swiss-Prot. Several methods exist that exploit database annotations to identify related proteins [14-17], however there has been no resource that very simply provides sets of FEPs annotated as having the same function in UniProtKB/Swiss-Prot in an easily-accessible format, with extensive coverage of multiple proteomes.

We have developed FOSTA (Functional Orthologues from SwissProt Text Analysis), which automates the process that one would perform manually to extract a family of FEPs from UniProtKB/Swiss-Prot. It considers UniProtKB/Swiss-Prot proteins for inclusion in groups of FEPs (FOSTA families) rooted around human proteins. It refines an initial candidate list of homologues on the basis of functional annotation similarity, to distinguish FEPs from functionally diverged homologues (FDHs). To assess functional annotation similarity, we employ simple text-mining techniques to compare UniProtKB/Swiss-Prot description fields.

## Results and discussion

Evaluating FOSTA is difficult because no gold-standard dataset exists. In addition, it is difficult to design an evaluation procedure to isolate the performance of FOSTA itself from the quality of the UniProtKB/Swiss-Prot annotations that FOSTA interprets. To assess the FOSTA *method*, we need to assess whether FOSTA is grouping proteins correctly into functionally equivalent groups given the functional annotations, rather than assessing whether the functional annotations are of sufficient detail to infer genuine functional equivalence. However, it is also very important to assess the latter, as FOSTA is dependent on the UniProtKB/Swiss-Prot annotations.

As such, FOSTA has been evaluated in three phases. The first involves manual interpretation of the results of several large protein families, some chosen at random, and some chosen as known problematic cases. This phase assesses how well FOSTA can interpret functional annotations, and infer functional equivalence compared with manual interpretation. The second phase benchmarks FOSTA against a fully manually annotated dataset, and a larger partially annotated dataset. This phase not only indicates whether FOSTA performs well, but also assesses whether the annotations are good enough to infer functional equivalence. The final phase of evaluation involves comparing UniProtKB/Swiss-Prot with InParanoid [10], a popular method for identifying orthologues.

FOSTA results are available at , by searching with the UniProtKB/SwissProt protein ID of interest. Results in this paper are for UniProtKB/Swiss-Prot version 53.0 (29th May 2007). The full set of FOSTA results may also be obtained in XML format, as can the results for a single human protein. A comprehensive help service is provided online, and updates will be performed every two months.

### An overview of FOSTA families

Before presenting the analysis of our method, we provide a brief description of the dataset. With a view to summarising the FOSTA dataset, we have calculated the 'UniProtKB/Swiss-Prot proteome coverage' for each species described in FOSTA. This has been calculated as *N*_*F*_*/N*_*SP*_, where *N*_*SP *_is the number of proteins from that species that are described in UniProtKB/Swiss-Prot (i.e., the size of the 'UniProtKB/Swiss-Prot proteome') and *N*_*F *_is the number of proteins from that species described in FOSTA. Therefore, a species which is fully represented in FOSTA with respect to its UniProtKB/Swiss-Prot proteome would have a UniProtKB/Swiss-Prot proteome coverage of 100%, while a species with none of its UniProtKB/Swiss-Prot proteins represented in FOSTA would have a UniProtKB/Swiss-Prot proteome coverage of 0%. Of the 11126 species represented in UniProtKB/Swiss-Prot version 53.0, just over half (52.73%) are not represented by FOSTA. This will in part be due to differing annotation formats of very remote species, but will also in part be due to distant species having very few proteins in common with the Human proteome. More positively, 2550 species (22.92%) are fully represented in FOSTA. Of course, many of these proteomes will be small, but nevertheless, it is encouraging that almost a quarter of UniProtKB/Swiss-Prot species are fully represented in FOSTA.

The most common family size is two: 25.48% (3793/14884) of FOSTA families with a non-human member have two members; this usually corresponds to an exclusively Human/Murine FOSTA family. These are not only the most well represented species in UniProtKB/Swiss-Prot version 53.0, they are also the most extensively and similarly annotated. 37.25% of FOSTA families (5545) have five or more members, and only 1.85% (275) have more than 50 members.

With respect to how FEP relationships are formed, most FOSTA families are formed exclusively using the protein prefix match, i.e., all members share the same protein prefix. However, 42.10% (6266/14884) of FOSTA families contain at least two different protein prefixes. Furthermore, of the 22 871 protein prefixes recorded in FOSTA, 5.42% are found to exist in more than one FOSTA family. This indicates that, although UniProtKB/Swiss-Prot protein prefixes are very often reliable, incorporating additional information derived from the description field is beneficial in identifying FEP relationships.

### HOX proteins

In the introduction, we discussed the family of HOX proteins as an example of a large family of proteins with multiple paralogues in each species. Here we assess the performance of FOSTA (and – by proxy – the quality of UniProtKB/Swiss-Prot annotations) when assigning the Zebrafish (*Danio rerio*) FEP to *Homo Sapiens *homeobox protein Hox-B7. There is a body of literature on the problem of elucidating HOX gene evolution, which is difficult in Zebrafish given the extensive polyploidy in its evolutionary history [18-20].

The BLAST search identifies 83 Zebrafish candidate FEPs and the filtering process assigns HXB7A_DANRE [Swiss-Prot:Q8AWY9] to the FOSTA family of HXB7_HUMAN [Swiss-Prot:P09629]. There are 24 Zebrafish FDHs that have higher sequence similarity to HXB7_HUMAN than the assigned FEP. These proteins, the FEP and the root human protein are listed in Table 1, along with their UniProtKB/Swiss-Prot annotations and their sequence identity to HXB7_HUMAN. It is clear that HXB7A_DANRE should be identified as the FEP given the similarity of its description to that of HXB7_HUMAN; this would be selected in a manual analysis of these candidates, despite its lower sequence identity.

**Table 1:** Zebrafish candidates for the FOSTA family of HXB7_HUMAN

**Protein**	**ID**	**Description**
HXB7_HUMAN	100	Homeobox protein Hox-B7; Hox-2C; HHO.C1
HXB7A_DANRE	54	Homeobox protein Hox-B7a; Hox-B7

HXA1A_DANRE	63	Homeobox protein Hox-A1a; Hox-A1
HXA3A_DANRE	68	Homeobox protein Hox-A3a
HXA4A_DANRE	65	Homeobox protein Hox-A4a; Zf-26; Hoxx4
HXA5A_DANRE	75	Homeobox protein Hox-A5a
HXA9B_DANRE	62	Homeobox protein Hox-A9b
HXB1A_DANRE	64	Homeobox protein Hox-B1a; Hox-B1
HXB1B_DANRE	64	Homeobox protein Hox-B1b; Hox-A1
HXB2A_DANRE	57	Homeobox protein Hox-B2a; Hox-B2
HXB3A_DANRE	67	Homeobox protein Hox-B3a; Hox-B3
HXB4A_DANRE	62	Homeobox protein Hox-B4a; Hox-B4; Zf-13
HXB5A_DANRE	75	Homeobox protein Hox-B5a; Hox-B5; Zf-21
HXB5B_DANRE	75	Homeobox protein Hox-B5b; Hox-B5-like; Zf-54
HXB6A_DANRE	78	Homeobox protein Hox-B6a; Hox-B6; Zf-22
HXB6B_DANRE	75	Homeobox protein Hox-B6b; Hox-A7
HXB8B_DANRE	60	Homeobox protein Hox-B8b; Hox-A8
HXC1A_DANRE	62	Homeobox protein Hox-C1a
HXC3A_DANRE	61	Homeobox protein Hox-C3a; Hox-114; Zf-114
HXC5A_DANRE	72	Homeobox protein Hox-C5a; Hox-C5; Hox-3.4; Zf-25
HXC6A_DANRE	63	Homeobox protein Hox-C6a; Hox-C6; Zf-61
HXC6B_DANRE	77	Homeobox protein Hox-C6b
HXC8A_DANRE	73	Homeobox protein Hox-C8a
HXD4A_DANRE	62	Homeobox protein Hox-D4a; Hox-D4
HXD9A_DANRE	65	Homeobox protein Hox-D9a; Hox-D9
HXDAA_DANRE	61	Homeobox protein Hox-D10a; Hox-D10; Hox-C10

**Protein**: The UniProtKB/Swiss-Prot ID; **ID**: The sequence identity of the **Protein **to HXB7_HUMAN;

**Description**: The UniProtKB/Swiss-Prot description (DE) field.

**Figure 1:**
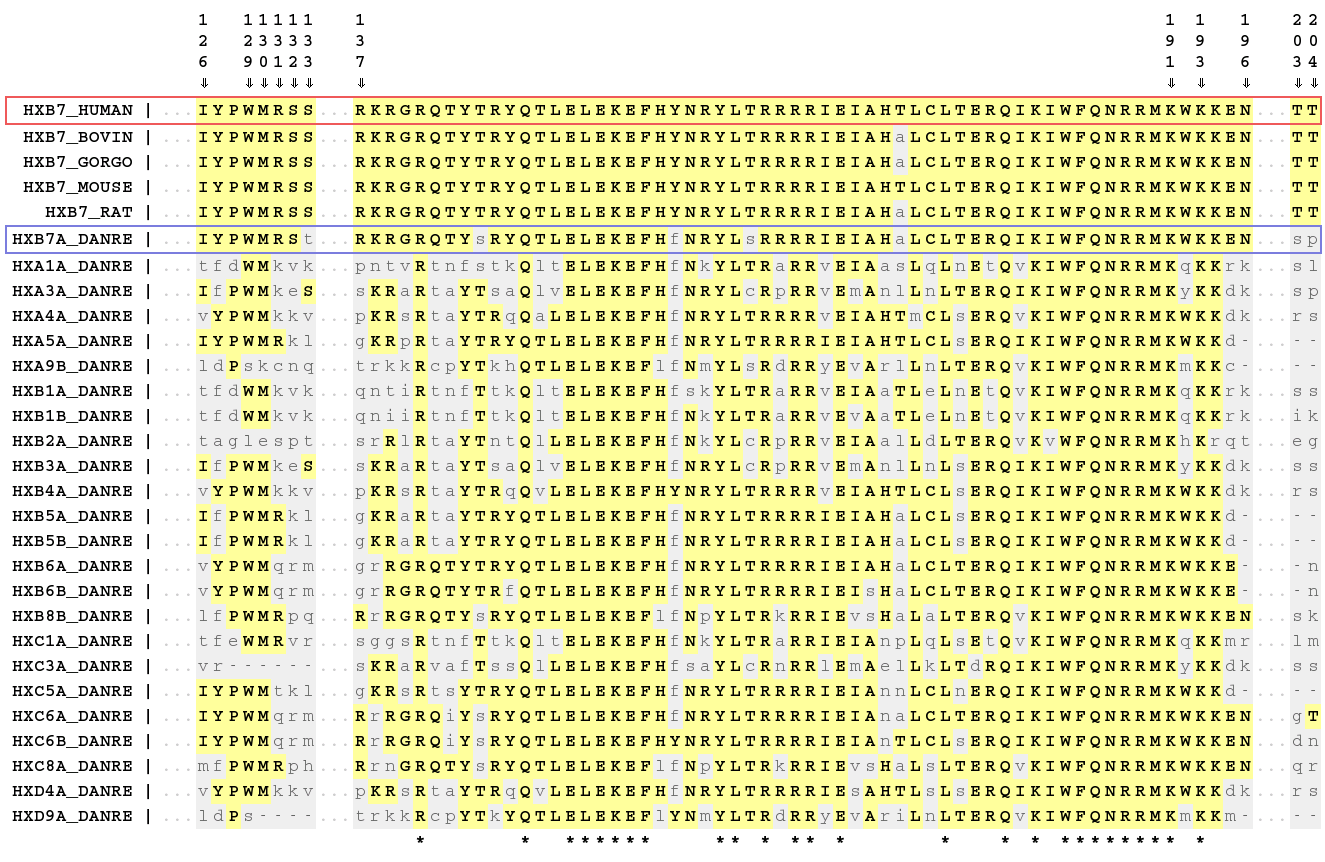
**Verifying the *Danio rerio *FEP of HXB7_HUMAN using annotated functional regions.** Residues identical to that of HXB7_HUMAN are in bold capitals and highlighted yellow, mismatching residues are non-captials and highlighted in light grey. The root human protein (HXB7_HUMAN) is indicated in the red box, and the assigned Zebrafish is highlighted in the blue box. The position relative to HXB7_HUMAN is given on the top line, and the asterisks on the bottom line highlight fully conserved columns.

Several sites of functional relevance have been identified for HXB7_HUMAN (Table 2). These functional sites have been extracted from UniProtKB/Swiss-Prot annotations and a mutagenesis study by Yaron *et al*. (2001). Figure 1 shows the alignment of the HXB7_HUMAN and the four confidently assigned FEPs with HXB7A_DANRE and the other fifteen *Danio rerio *candidates in the functionally relevant areas. Despite *globally *having the lowest sequence identity to HBX7_HUMAN of all the Zebrafish proteins shown in Figure 1, it is clear that HXB7A_DANRE has the highest conservation at functionally critical sites. Across residues 126 to 133, HXB7A_DANRE only differs from HXB7_HUMAN at the position of a putative PBX binding site, unlike the other *Danio rerio *proteins which all differ in a known sequence motif. The homeobox region (which also includes crosslinking sites) is highly conserved across all of the Zebrafish proteins, and again, conservation is highest in HXB7A_DANRE. None of the Zebrafish proteins show conservation at residues 203 and 204, which describe a putative CKII target site [3]. It is possible that this functional site has been wrongly predicted; however, this is unlikely as it is absolutely conserved across the five mammalian species. It is more likely that this region is no longer functional in the *Danio rerio *lineage, or that this is a recently acquired functionality in the mammalian clade.

**Table 2:** Functional sites in HXB7_HUMAN

**Functional site**	**Location**	**Reference**
DNA binding (homeobox)	137 – 197	UniProtKB/Swiss-Prot FT/DNA_BIND annotation
Crosslink (glycyl lysine isopeptide)	191 & 193	UniProtKB/Swiss-Prot FT/CROSSLNK annotation
Motif (Antp-type hexapeptide)	126 – 131	UniProtKB/Swiss-Prot FT/MOTIF annotation
Hypothesized binding to PBX	129 – 130	Yaron *et al*. [3]
Putative CKII target	132 – 133	Yaron *et al*. [3]
Putative CKII target	203 – 204	Yaron *et al*. [3]

**Functional site**: a description of the functional site; **Location**: the residue number in HXB7_HUMAN;

**Reference**: The source of the annotation.

### A solved annotation problem: PROC_HUMAN

The UniProtKB/Swiss-Prot ID consists of a protein name followed by an underscore and the species name. It was our initial assumption that the protein name part of the ID was a unique name used to label FEPs [21]. However, while analysing human protein C (PROC_HUMAN) using UniProtKB/Swiss-Prot version 50.6, it was evident that this approach was unreliable. The 'PROC' prefix was in forty different species to describe three different proteins: Procalin in one species (PROC_TRIPT, [Swiss-Prot:Q9U6R6]), protein C in 11 species (e.g., PROC_HUMAN, [Swiss-Prot:P04070]), and pyrroline-5-carboxylate reductase in the remaining 28 species (e.g., PROC_ECOLI, [Swiss-Prot:P0A9L8]) (UniProtKB/Swiss-Prot version 53.0). A previous version of FOSTA was successful in correctly assigning only true examples of protein C to the FEP group, and analysis of human pyrroline-5-corboxylate reductase results highlighted the inconsistencies in UniProtKB/Swiss-Prot naming conventions (data not shown).

Several of the FEPs in the FOSTA families of P5CR1_HUMAN (pyrroline-5-carboxylate reductase 1) and PROC_HUMAN (protein C) have had multiple protein prefix changes. However, after notifying UniProtKB/Swiss-Prot of the discrepancies, all the misnamed proteins were corrected for the release of UniProtKB/Swiss-Prot v51.2: pyrroline-5-carboxylate reductase proteins prefixed with PROC or PROH are now prefixed with P5CR or P5CR1 and PROC_TRIPT (procalin) is now called PRCLN_TRIPT.

UniProtKB/Swiss-Prot makes no guarantee that the protein prefix is a unique identifier, instead describing it as a 'mnemonic code', but it is stressed that work is ongoing to standardize protein nomenclature:

*"Ambiguities regarding gene/protein names are a major problem in the literature and it is even worse in the sequence databases which tend to propagate the confusion... UniProt is constantly striving to further standardize the nomenclature for a given protein across related organisms"* ().

Although this standardisation is discussed only with respect to protein names, and not the protein prefix elements of the UniProtKB/Swiss-Prot IDs, it is evident from the timings of prefix updates for protein C and pyrroline-5-carboxylate reductase proteins since UniProtKB/Swiss-Prot version 53.0 that UniProtKB/Swiss-Prot does aim to standardize protein prefixes. If this ID was used consistently across all proteins in UniProtKB/Swiss-Prot there would be no need for FOSTA.

### Manual analysis of five protein families

To evaluate FOSTA, a manual analysis of five protein families was carried out. The focus was the description fields, and whether the description matches by FOSTA were appropriate. The first was trypsin-1 (TRY1_HUMAN, [Swiss-Prot:P07477]), which was chosen because it belongs to the large serine protease family of proteins. The remaining four – glucose-6-phosphate isomerase (G6PI_HUMAN, [Swiss-Prot:P06744]), aminopeptidase N (AMPN_HUMAN, [Swiss-Prot:P15144]), ATP-dependent RNA helicase DDX51 (DDX51_HUMAN, [Swiss-Prot:Q8N8A6]) and protoheme IX farnesyltransferase (COX10_HUMAN, [Swiss-Prot:Q12887]) – were chosen at random. The results are summarised here (more detailed discussion is available in the Additional Files). All results are available by searching for the root protein at .

Fifteen of the FEPs identified for TRY1_HUMAN are clearly trypsin molecules (the other three are closely related serine proteases). It is notable that all five questionable assignments are derived from insect species; it may be that trypsin genes have diverged and/or duplicated in insect species, or it may be that the naming conventions for trypsin proteins are quite different in insect species. To assign FEPs to AMPN_HUMAN, FOSTA is required to discriminate between multiple different families of aminopeptidases. Of the twenty four FEPs that are identified, only seven would require further investigation to confirm the pairing and the same requirement would apply to manual analysis of the annotations. DDX51_HUMAN belongs to a large family of 'DEAD box helicases'; identifying functional equivalence in such a large family of proteins is a difficult task. Nevertheless, three of the five fully sequenced proteins identified by FOSTA are correct (according to the manually confirmed UniProtKB/Swiss-Prot family/domain classifications given for the DEAD box helicases); the remaining two are from different subfamilies. The results for the two remaining proteins – G6PI_HUMAN and COX10_HUMAN – are very robust.

The vast majority of the FEP assignments considered in this section are correct, and no results are clearly wrong. Where results are questionable, it is not clear whether differing naming conventions across species are hindering the identification of the true FEP, or whether protein function has diverged in other species. However, it should again be stressed that a manual analysis of UniProtKB/Swiss-Prot entries for these families is no more effective than FOSTA.

### Further Benchmarking

Evaluation of FEP extraction is difficult as no gold standard, large, manually annotated dataset of one-to-one FEP pairings is available. It is important to note that FOSTA is simply an automated assimilation of existing information that has been curated; it is not a method for inferring functional relationships from low level data. In other words, rather than find *novel *functional relationships, FOSTA aims to extract functional relationships described in curated UniProtKB/Swiss-Prot annotation.

Nevertheless, we have benchmarked the FOSTA results against two datasets: the large, partially manually annotated PIRSF dataset [22] and a refinement of Hulsen *et al*.'s manually curated dataset of six protein families that has been used previously to evaluate orthologue identification methods [23] (the refinement procedure identified the true one-to-one pairings in the one-to-many sets).

FOSTA is designed to be conservative in the FEP assignments it makes: it is more important to minimise the number of false positives than to minimise the number of false negatives. Therefore, the most appropriate performance statistic with which to evaluate FOSTA is the positive predictive value (PPV): the proportion of positive predictions that are correct, *TP/*(*TP *+ *FP*). To provide an overall performance statistic, we also report the Matthews Correlation Coefficients (MCC). Further performance statistics (sensitivity and specificity) are included in the Additional Files.

#### PIRSF evaluation

The Protein Information Resource (PIR) is a widely used, publicly available resource, and is part of the UniProtKB consortium. With a view to the standardization of accurate propagation of protein annotations, PIR has developed the PIRSF (PIR super family) classification system for UniProtKB proteins [22]. However, unlike FOSTA it does not identify FEPs as it contains many-to-many orthologous pairings.

FOSTA was benchmarked against all one-to-one orthologous relationships between UniProtKB/Swiss-Prot proteins that are listed in PIRSF families as 'regular' members ('associate' members can be alternative splice variants, which should not be FEPs), at all four levels of curation, where PIRSF families with a curation status of *'Full/Desc' *have the highest level of manual curation, and families with a curation status of *'None' *have not been manually curated.

It is evident from Table 3 that FOSTA performs extremely well on the PIRSF protein families according to the PPV and specificity metrics that are particularly important. However, it also demonstrates reasonably high sensitivity and very high MCC scores.

**Table 3:** Benchmarking FOSTA against the PIRSF dataset

**Set**	**Families**	**Pairings**	**Basic statistics**				**Evaluation statistics**	
			**TP**	**FP**	**TN**	**FN**	**PPV**	**MCC**
**A**	122	2127	1744	2	3717	383	99.89	0.86
**B**	1095	18865	12967	23	34656	5898	99.82	0.77
**C**	474	11221	9146	62	11819	2075	99.33	0.83
**D**	339	5287	3674	16	4938	1613	99.57	0.72

**N**	1691	32213	23857	87	50192	8356	99.64	0.79
*	2020	37500	27531	103	55130	9969	99.63	0.79

**Set ID**: the identifier for each curation set [A='Full/Desc.', B='Full', C='Preliminary', D='None', N=aNnotated (A+B+C), * = All (N+D)]; **Curation string**: the string that defines the curation set; **Families**: the number of discrete protein families in the curation set; **Pairings**: the number of discrete pairings across all families to be tested in FOSTA; **Basic statistics**: the basic counts of true positives (TP), false positives (FP), true negatives (TN), false negatives (FN); **Evaluation statistics**: the **PPV **(positive predictive value, *TP/*(*TP *+ *FP*)), and the **MCC **(Matthews Correlation Coefficient), all rounded to 2dp

#### Refined Hulsen evaluation

Hulsen *et al*. [23] recently evaluated the performance of several orthologue identification methods: BBH (bidirectional best hit), Inparanoid [10], KOG [12], OrthoMCL [24], PhyloGeneticTree [25] and Z 1 hundred (estimating statistical significance of alignment scores). The benchmarking included comparison with manually annotated 'true-orthologue' (TO) pairs of six protein families. For human-mouse (*Homo sapiens *and *Mus musculus*) pairings, the protein families used were the homeobox proteins (HOX), haemoglobins (HBB), and Sm and Sm-like proteins (SMm). For human and worm (*Caenorhabditis elegans*) TO pairs, the families used were nuclear receptors (NR), toll-like receptors (TLR), and Sm and Sm-like proteins (SMc).

These methods all aim to identify orthologues and do not consider functional equivalence. Since they have different goals, it is not possible to compare FOSTA directly with the methods evaluated by Hulsen *et al*., but we can evaluate FOSTA using a subset of the TO data.

The TO dataset supports many-to-many orthologous pairings where a human protein can map to one or more proteins in another species, and vice versa. To evaluate FOSTA, these data were manually refined to include only those TO pairings that can be confidently identified as true one-to-one orthologous pairings, where *both *proteins can be mapped to UniProtKB/Swiss-Prot (c.f. Refined and TO in Table 4). This refinement process removes the TLR dataset from the analysis, as no definitive one-to-one orthologous pairings could be identified through manual inspection.

**Table 4:** Benchmarking FOSTA against the refined Hulsen *et al*. dataset

**Protein family**	**Refined**	**(TO)**	**Basic statistics**				**Evaluation statistics**	
			**TP**	**FP**	**TN**	**FN**	**PPV**	**MCC**
**HBB**	2	(9)	2	0	17	0	100.00	1.00
**HOX**	30	(41)	30	0	3853	0	100.00	1.00
**SMm**	12	(17)	12	0	22	0	100.00	1.00
**SMc**	6	(6)	6	0	5	0	100.00	1.00
**NR**	4	(29)	1	1	327	3	50.00	0.35

**All**	54	(102)	51	1	4224	3	98.08	0.96

**Protein family**: the protein family being examined; **TO pairings**: the number of TO pairs in the Hulsen dataset (including many-to-many orthologous pairings and non-UniProtKB/Swiss-Prot proteins); **Refined pairings**: the number of one-to-one TO pairings tested after refinement of Hulsen TO dataset; **Basic statistics**: the basic counts of true positives (TP), false positives (FP), true negatives (TN), false negatives (FN); **Evaluation statistics**: the **PPV **(positive predictive value, *TP/*(*TP *+ *FP*)), and the **MCC **(Matthews Correlation Coefficient), all rounded to 2dp)

The results are summarised in Table 4. FOSTA demonstrates perfect performance in the HBB, HOX, SMm and SMc families, identifying all refined true-orthologue pairings, and avoiding any false positive FEP assignments.

However, FOSTA identified only one of the refined human/worm nuclear receptor (NR) TO pairs (NHR67_CAEEL). On closer inspection, it is evident that the three failures of FOSTA in the NR dataset are a result of widely varying formats of the UniProtKB/Swiss-Prot description field across the two species; for example, the *Homo sapiens *proteins tend to be named as "Nuclear receptor subfamily X group Y member Z" proteins, whereas the *Caenorhabditis elegans *proteins are named as "Nuclear hormone receptor family member nhr-N" proteins. These primary protein names or descriptions are defined by the species-specific annotation communities (for example, Human Genome Nomenclature Committee, FlyBase and Caenorhabditis Genetics Centre/Wormbase for *Homo sapiens*, *Drosophila melanogaster *and *Caenorhabditis elegans *respectively) with additional synonyms obtained by UniProtKB/Swiss-Prot from the literature. Therefore, we cannot strictly attribute the lack of annotation consistency to problems in UniProtKB/Swiss-Prot, as UniProtKB/Swiss-Prot is merely reflecting the differing practices of the annotation communities and the content of the literature. Nevertheless, the lack of consistent description field formatting within UniProtKB/Swiss-Prot limits the extent to which text-mining methods such as FOSTA can exploit the data.

It is encouraging to note that FOSTA makes only one false positive assignment in the refined Hulsen dataset. Furthermore, FOSTA does not eliminate any of the one-to-one TO pairs: where a FEP relationship is missed, the TO is retained as a FDH, indicating that our BLAST threshold is not too conservative.

### Comparison with Inparanoid

Inparanoid is a well-known method of constructing sets of orthologous proteins [10]. It uses BBH (best bi-directional hit) pairs in different species as a 'seed' around which a cluster of orthologues can be formed. Other orthologues – or specifically other inparalogues – can be added to this pairing if they are more similar to one of the seed orthologues than they are to any other protein in another species.

Inparanoid does not perform the same task as FOSTA. FOSTA is specifically interested in identifying functionally similar proteins whereas Inparanoid is more interested in identifying the phylogenetic relationships between proteins in different species. As such, where Inparanoid detects one-to-one orthologous pairs, the results will be largely complementary, but need not be identical. We therefore cannot 'benchmark' against Inparanoid: it is not the gold standard dataset. However, by identifying one-to-one orthologous pairings in the Inparanoid dataset that FOSTA rejects as FEPs, we have a dataset of proteins that we can consider as more difficult test cases. For convenience, we will refer to one-to-one orthologous pairings in the Inparanoid dataset as 'Inparanoid pairs' or IPs, and IPs that FOSTA does not consider functionally equivalent as 'rejected IPs'.

Columns 1–3 in Table 5 describe how many IPs from each species were successfully mapped to UniProtKB/Swiss-Prot IDs, and therefore how many IPs from each species can be compared to FOSTA. 27069 IPs were extracted from Inparanoid v6.1, of which 26073 (96.32%) are verified by FOSTA. Of the 996 IPs that are not found in FOSTA, 125 are rejected in favour of another UniProtKB/Swiss-Prot protein from the non-human species (these IPs will be described as 'conflicting' IPs); in the remaining 871 IPs, FOSTA fails to assign any FEP from the non-human species to the human protein ('rejected' IPs).

**Table 5:** Comparing FOSTA with Inparanoid

**Code**	**Species**	**Pairs**	**Matches**	**Mismatches**	**% match**	**Overlooked**	**Rejected**
APIME	*Apis mellifera*	1	1	0	100.00%	-	-
BOSTA	*Bos taurus*	3508	3451	57	98.38%	1	56
CANFA	*Canis familiaris*	533	520	13	97.56%	1	12
CIOIN	*Ciona intestinalis*	6	5	1	83.33%	0	1
DANRE	*Danio rerio*	1246	1192	54	95.67%	21	33
DICDI	*Dictyostelium discoideum*	85	69	16	81.18%	0	16
DROME	*Drosophila melanogaster*	878	712	166	81.09%	14	152
DROPS	*Drosophila pseudoobscura*	73	67	6	91.78%	0	6
GALGA	*Gallus gallus*	1360	1297	63	95.37%	12	51
GASAC	*Gasterosteus aculeatus*	1	1	0	100.00%	-	-
MACMU	*Macaca mulatta*	214	207	7	96.73%	0	7
MONDO	*Monodelphis domestica*	22	21	1	95.45%	0	1
MUSMU	*Mus musculus*	12063	11960	103	99.15%	18	85
ORYSA	*Oryza sativa*	1	0	1	0.00%	0	1
PANTR	*Pan troglodytes*	412	408	4	99.03%	1	3
RATNO	*Rattus norvegicus*	5076	5005	71	98.60%	6	65
SACCE	*Saccharomyces cerevisiae*	1213	787	426	64.88%	49	377
TETNI	*Tetraodon nigroviridis*	6	6	0	100.00%	-	-
XENTR	*Xenopus tropicalis*	371	364	7	98.11%	2	5

-	All species	27069	26073	996	96.32%	125	871

**Code**: The species code as used by Inparanoid; **Species**: The full species name; **Pairs**: The number of one-to-one orthologue pairs described by Inparanoid between **Species **and Human; **Matches**: The number of one-to-one Inparanoid orthologue pairs (IPs) that are also found by FOSTA; **Mismatches**: The number of IPs pairs that are *not *found by FOSTA; **% match**: The percentage of IPs that are also found by FOSTA; **Overlooked**: The number of IPs where FOSTA assigns a different protein from the **Species **to the FOSTA family of the human protei; **Rejected**: The number of IPs where FOSTA does not assign any protein from the **Species **to the FOSTA family of the human protein.

These datasets have been further 'cleaned' to remove those IPs that either (i) cannot be found by FOSTA or (ii) are clearly correct in FOSTA. 43 of the 125 conflicting IPs appear to be wrong in Inparanoid, since the FEP that FOSTA assigns matches the human protein confidently using the protein prefix match (manual analysis confirms this conclusion); for example, FOSTA identifies ADA2A_RAT as a FEP of ADA2A_HUMAN, while Inparanoid assigns ADA2C_RAT as the FEP in *Rattus norvegicus*. A further five of the conflicting IPs appear to be wrong as the non-human protein chosen by Inparanoid is assigned as a FEP using a protein prefix match elsewhere in FOSTA. For example, Inparanoid assigns SPDYA_MOUSE to the FOSTA family of SPDYC_HUMAN, while FOSTA assigns SPDYB_MOUSE and confidently assigns SPDYA_MOUSE to the FOSTA family of SPDYA_HUMAN. 36.74% of the 871 rejected IPs cannot be identified by FOSTA: 26.98% are not found using a BLAST threshold of 10^-2 ^and 1.15% involve short human proteins that FOSTA does not analyse (see Methods). A further 75 rejected Inparanoid assignments are found to be wrong: FOSTA assigns the non-human protein elsewhere on the basis of a protein prefix match.

This leaves a 'clean' dataset of 77 overlooked IPs and 551 rejected IPs. In a random sample of ten of the overlooked IPs (see Additional Files), three FOSTA assignments and one Inparanoid assignment appear to be correct. There is not enough evidence in the six remaining overlooked IPs to ascertain which assignment might be correct; however, four of the six remaining IPs are flagged as less reliable sequence matches by FOSTA and could therefore be removed from the dataset.

A random sample of 28 IPs (approximately 5%) were selected from the rejected dataset (see Additional Files). Note that the IPs described in this dataset are not necessarily correct; however we can use the IPs as examples of difficult test cases, and hypothesize why FOSTA might not identify them. Most of the IPs are rejected by FOSTA due to uninformative or sparsely annotated DE fields. A significant number arise from large, densely populated protein families in which functional relationships are hard to elucidate.

Only two highlight where the FOSTA functional match methodology may lack sensitivity; these are shown in Table 6. The first example – CC45L_HUMAN/CDC45_YEAST – suggests that mapping from acronyms to long forms and vice versa may be valuable in future version of FOSTA; in this example, CDC would be extended to 'Cell division control'. In the second FGF17_HUMAN/FG17B_DANRE example, some flexibility in names and numbers used by the matching machinery would lead to these two proteins being identified as FEPs. However, introducing such additional flexibility without careful consideration would increase the likelihood of false positives being introduced into the FOSTA dataset.

**Table 6:** Example insensitivities in the FOSTA functional match methodology

**Mapping to/from acronyms and long forms**	
CC45L_HUMAN	CDC45-related protein; PORC-PI-1; Cdc45
CDC45_YEAST	Cell division control protein 45
**Allowing for slign variations in names and numbers**	
FGF17_HUMAN	Fibroblast growth factor 17 precursor; FGF-17
FG17B_DANRE	Fibroblast growth factor 17b precursor; FGF-17b

The Inparanoid data are mapped to UniProtKB/Swiss-Prot using UniProtKB/Swiss-Prot cross-references. Unfortunately using the UniProtKB/Swiss-Prot cross-references to map from the Inparanoid ENSEMBL protein IDs to UniProtKB/Swiss-Prot sequences results in a biased dataset: the UniProtKB/Swiss-Prot sequences with explicit cross-references are likely to be well-annotated. Nevertheless, it is reassuring that where the Inparanoid dataset does identify one-to-one pairings between UniProtKB/Swiss-Prot proteins, FOSTA confirms 95.99% in a large dataset (27 069 protein pairs) in a wide variety of species (*Apis mellifera*, *Bos taurus*, *Conis familiaris*, *Ciona intestinalis*, *Danio rerio*, *Dictyostelium discoideum*, *Drosophila melanogaster*, *Drosophila pseudoobscura*, *Gallus gallus*, *Gasterosteus aculeatus*, *Macaca mulatta*, *Monodelphis domestica*, *Mus musulus*, *Oryza sativa*, *Pan troglodytes*, *Rattus norvegicus*, *Saccharomyces cerevisiae*, *Tetraodon nigroviridis *and *Xenopus tropicalis*). Further, there are no FOSTA assignments that appear spurious.

## Conclusion

FOSTA is a novel method that extracts functionally equivalent proteins (FEPs) from the UniProtKB/Swiss-Prot database by 'reading' the UniProtKB/Swiss-Prot annotations. As such, it is a grouping of UniProtKB/Swiss-Prot proteins that are annotated similarly. We take advantage of the fact that UniProtKB/Swiss-Prot annotations are the result of many hours of manual annotation, and should encapsulate all knowledge available to the annotator at the time.

Since FOSTA simply assimilates existing annotations, it is difficult to separate the performance of the FOSTA *method*, from the quality and consistency of annotations in UniProtKB/Swiss-Prot. Manual analysis of eight FOSTA families, two benchmarking evaluations and a comparison to the popular but quite different Inparanoid method indicate that FOSTA performs well and that UniProtKB/Swiss-Prot annotations are generally of high quality. In addition to providing researchers with genuine FEP families for tasks such as studying sequence conservation, FOSTA could be used to provide datasets to evaluate function prediction methods.

Given the methodology, FOSTA has a few limitations. Firstly, FOSTA is clearly dependent on UniProtKB/Swiss-Prot annotations. Any method based on database annotations is potentially problematic as it relies on possibly mistaken, incomplete, inconsistent, ambiguous or outdated information. However, the UniProtKB/Swiss-Prot database is considered to be the gold standard for protein annotation (our benchmarking results reflect that the annotations are indeed very reliable), and annotations are constantly revised (for example, 210454 annotation revisions were made between release UniProtKB/Swiss-Prot v52.0 and UniProtKB/Swiss-Prot version 53.0 ). The continuous revision of UniProtKB/Swiss-Prot with the regular update of FOSTA ensures that FOSTA FEP assignments can only improve in parallel with UniProtKB/Swiss-Prot. Secondly, clearly only proteins described in UniProtKB/Swiss-Prot can be assigned to FOSTA families. Given that UniProtKB/Swiss-Prot is growing at an exponential rate () and that it is the aim to include all proteins in UniProtKB/Swiss-Prot, this limitation is not considered significant.

If FOSTA cannot discriminate between two candidate FEPs on the basis of function, it will choose the candidate with the higher sequence identity to the root; only 6047 of FEP assignments (5.00%) are made on this basis. Any sequence matching is undesirable, as high sequence similarity does not necessarily imply precise functional equivalence. It may be avoided if more sensitive information extraction methods could be implemented to improve functional discrimination. UniProtKB/Swiss-Prot keywords and GO terms may have some value, but these tend to be at a higher level of annotation and are unlikely to improve discrimination of very detailed functional information. While automatic acronym resolution and character-based fuzzy matching might improve performance, more sophisticated natural language processing methods [26] would not be expected to help, as the text being examined is simply a list of nouns. Alternatively, a more sensitive sequence matching protocol could be implemented where annotated functional residues, or a consensus profile of FEPs already assigned with high confidence could be used, rather than the whole sequence which may be misleading. Furthermore, a vocabularly mapping acronyms to their long forms and vice versa, and/or mapping between known synonyms may improve the functional comparison step.

FOSTA's requirement for one-to-one FEP relationships may also be viewed as a limitation. However, we consider this to be justified. Consider the protein *X *in species *A *that has two homologues *Y*_1 _and *Y*_2 _in species *B*. If *Y*_1 _and *Y*_2 _are both homologous to *X*, one must have been derived via a gene duplication event. Gene duplication is a mechanism for functional divergence, and we therefore argue that one of either *Y*_1 _or *Y*_2_, most likely (though not necessarily) the one with the poorer sequence identity to *X*, has acquired novel, or lost existing, functionality (or is in the process of doing so), and should not be selected as a FEP.

Currently, FOSTA roots families around human proteins because we were interested in identifying FEPs to human proteins, to examine human disease. 58.36% (169523 of 290484) of UniProtKB/Swiss-Prot proteins are not assigned to a FOSTA family in the current version. Using the median size of a FOSTA family (87), we can estimate that another 1949 families will be formed if FOSTA were to cluster around non-human proteins. We propose that a future version of FOSTA will root FOSTA families around decreasingly well defined (in terms of proteome coverage and functional annotation in UniProtKB/Swiss-Prot) species, until all proteins are assigned to a FOSTA family. While we intend to address this in future versions, it must be noted that human proteins are the most thoroughly annotated, and it is unclear whether proteins from other organisms will be annotated well enough to identify functional equivalencies across species.

More generally, a controlled vocabulary for UniProtKB/Swiss-Prot description fields which would allow description of all proteins across all species, would allow text mining to make more reliable hypotheses. This might be implemented as a second, computer-friendly DE-type field, keeping the existing descriptions for human inspection. In addition, it would be desirable to move some information from the description field into separate tags in the UniProtKB/Swiss-Prot flatfile format; for example, flags for fragmented or hypothetical sequences. Given the size of UniProtKB/Swiss-Prot (UniProtKB/Swiss-Prot version 53.0 contains 290484 proteins), the resource must expect to be interrogated computationally, more so with every new release. Any effort from UniProtKB/Swiss-Prot to make its contents more computationally accessible would be valuable.

As stated above, a guarantee of unique UniProtKB/Swiss-Prot protein ID prefixes for equivalent proteins in different species would preclude the need for hypotheses to be drawn by software such as FOSTA. It is clear that the UniProtKB/Swiss-Prot team are making efforts to standardise such annotations across species (); however it is also clear that some efforts are not yet propagated fully across all relevant proteins and species. As stated above, the protein C/pyrroline-5-carboxylate reductase case described above has since been rectified by the UniProtKB/Swiss-Prot annotators.

It is clear that not only is the automatic extraction of FEPs a surprisingly difficult problem, but that it is also very difficult to evaluate these methods. The evaluation that was performed not only demonstrated that FOSTA performs well, but also that the vast majority of UniProtKB/Swiss-Prot annotations considered are of high quality. This provides further justification of an annotation-based methods such as FOSTA, and indicates that any concern about FOSTA's dependence on annotations need not be over-emphasized. In addition, we expect that FOSTA will improve with every revision of UniProtKB/Swiss-Prot.

## Availability and requirements

• Project name : FOSTA

• Project homepage : 

• Operating system : Web-based (runs under Linux)

• Programming language : Perl/SQL

• Other requirements : none for web use (uses PostgreSQL)

• License : N/A for web use (license negotiable for local installation)

• Any restrictions to use by non-academics : none for web use (license negotiable for local installation)

## Methods

### FOSTA

Carrying out the task of identifying functionally equivalent proteins for an individual case is relatively trivial: candidate FEPs are identified on the basis of sequence similarity and the FEP for each species is then identified by reading the annotations manually. FOSTA is designed to simulate this simple behaviour on a databank-wide scale, by examining UniProtKB/Swiss-Prot annotations to extract information about equivalences across different species.

**Figure 2:**
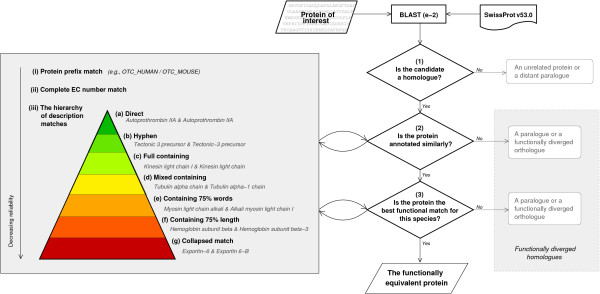
The FOSTA filtering process: homologues are identified by BLAST-ing against the UniProtKB/Swiss-Prot database (filtering stage (1)); these are then filtered to retain only those with similar function (filtering stage (2)); finally one protein per species (the FEP, or functionally equivalent protein) is chosen using a hierarchy of functional matches to eliminate functionally diverged homologues (FDHs) (filtering stage (3)).

As input, FOSTA takes an entire UniProtKB/Swiss-Prot release; results presented here are based on UniProtKB/Swiss-Prot version 53.0. FOSTA roots families of FEPs (FOSTA families) around human proteins using the three stage filtering processes shown in Figure 2. Candidates rejected at filtering stages (2) and (3) are retained and recorded as functionally diverged homologues (FDHs).

#### Stage (1) : The sequence filter

The first stage identifies sequence homologues using a BLAST [27] e-value threshold of 10^-2^. This list of candidate FEPs is then refined using the following two filters.

#### Stage (2) : The functional filter

This stage aims to 'read' the UniProtKB/Swiss-Prot annotations. The homologues obtained in the previous stage are filtered on function using information from the UniProtKB/Swiss-Prot 'Description' (DE) field and the UniProtKB/Swiss-Prot ID itself. Each homologue identified by the BLAST search will survive the functional filter if it matches the root protein in at least one of three levels: (i) by the protein element of the UniProtKB/Swiss-Prot ID, (ii) by an EC number, or (iii) by matching synonyms at further multiple levels of specificity from the DE field. All text comparisons are case insensitive. The DE field text matches compare synonyms at seven levels of specificity: (a) a 'direct' match, where the two proteins share an intact synonym; (b) a 'hyphen' match, where the proteins share a synonym after hyphen placement is mirrored across both strings; (c) a 'full containing' match, where one synonym is completely contained within another; (d) a 'mixed containing' match, where one synonym is contained within another synonym, but the words need not be in the same order; (e) a 'containing 75% words' match, where 75% of the words of the shorter synonym are also in the longer synonym; (f) a 'containing 75% length', where 75% of the words in terms of *length *of the shorter synonym are also in the longer synonym; (g) a 'collapsed' match, where one synonym is a substring of another, after spaces and punctuation have been removed. Full details are available on the website. The level (i) protein prefix match is considered the most reliable functional match (given that we know all candidates are homologues) and the level (iii) description match the least reliable functional match. Within the description field match, reliability reduces from (a) the direct match to (g) the collapsed match. Although the choice of the 75% threshold is somewhat arbitrary, it is unlikely that false matches will be made, as all candidates have already been screened for homology.

#### Stage (3) : The FEP filter

If a protein survives both the sequence and functional filtering stages, it is either the FEP for that species or a homologue which has undergone some (small) degree of functional divergence. To eliminate the FDHs, only the best functional match from each species (as defined by the functional match reliability hierarchy described in stage (2) above, and in the match hierarchy pyramid shown in Figure 2, is assigned to the FOSTA family. If two or more proteins cannot be discriminated *functionally *– i.e., their annotations match at the same level of specificity to those of the root human protein – the protein with the highest sequence identity is chosen (given that, as discussed in the Introduction, our objective is to define *one-to-one *functionally equivalent protein relationships). Note that sequence identity is used only as a last resort as highest sequence identity does not necessarily indicate functional equivalence even amongst close homologues [28,29].

Full details of the method are available at . FOSTA was run on ≈10 dual-core Opteron 270 2MHz CPUs using the Sun Grid Engine. Wall-clock run time is approximately eleven hours. All code was implemented in Perl using the DBI interface to the PostgreSQL relational database. Figures were generated using xfig and HTML.

### Extracting data from Inparanoid

The XML formats of Inparanoid v 6.1 were obtained by ftp from  and parsed using the Perl module XML::DOM. All Human/X one-to-one orthologues described by Inparanoid (IPs or Inparanoid pairs) were extracted. There were fifteen species in which no IPs were found (*Aedes aegypti*, *Anopheles gambiae*, *Arabidopsis thaliana*, *Caenorhabditis briggsae*, *Caenorhabditis elegans*, *Caenorhabditis remanei*,*Candida glabrata*, *Cryptococcus neoformans*, *Debaryomyces hansenii*, *Entamoeba histolytica*, *Escherichia coliK12*, *Kluyveromyces lactis*, *Schizosaccharomyces pombe*, *Takifugu rubripes *and *Yarrowia lipolytica*), leaving nineteen species with at least one IP to compare with FOSTA.

As FOSTA groups UniProtKB/Swiss-Prot pairings, all extracted IPs had to be mapped to UniProtKB/Swiss-Prot. Inparanoid proteins are described using various database IDs, including Ensembl (*Apis mellifera*, *Bos taurus*, *Canis familiaris*, *Ciona intestinalis*, *Gallus gallus*, *Gasterosteus aculeatus*, *Macaca mulatta*, *Monodelphis domestica*, *Pan troglodytes*, *Rattus norvegicus*, *Tetraodon nigroviridis*, *Xenopus tropicalis*), TAIR (*Arabidopsis thaliana*), Zfin (*Danio rerio*), Dictybase (*Dictyostelium discoideum*), Flybase (*Drosophila melanogaster *and *Drosophila pseudoobscura*), MGI (*Mus musculus*), Gramene (*Oryza sativa*) and Sanger (*Saccharomyces cerevisiae*). All relevant cross-references were extracted from UniProtKB/Swiss-Prot version 53.0; any contradicting or multiple cross-references (e.g., X → Y, X → Z) were not used.

## Authors' contributions

LEMM implemented and ran FOSTA, built the web server and drafted the paper. ACRM conceived and directed the project, and finalised the manuscript.

## Supplementary Material

Additional file 1 **Additional analysis of the FOSTA method.** File contains additional analysis and benchmarking of the FOSTA method using two datasets of functionally equivalent proteins and five protein families Click here for file
