# Supplementary material for: Automatically extracting functionally equivalent proteins from SwissProt
Source: BMC Bioinformatics. 2008 Oct 6;9:418. doi: 10.1186/1471-2105-9-418 (PMC2576269; doi:10.1186/1471-2105-9-418)
Supplement: Additional file 1 — Additional analysis of the FOSTA method. File contains additional analysis and benchmarking of the FOSTA method using two datasets of functionally equivalent proteins and five protein families [file 1471-2105-9-418-S1.pdf]

Supplementary materials

Manual analysis of five protein families: extended discussion

To evaluate FOSTA, a manual analysis of five protein families was carried out. The focus was the description fields, and whether the description matches by FOSTA were appropriate. The first was trypsin-1 (TRY1_HUMAN, [Swiss-Prot:P07477]), which was chosen because it belongs to the large serine protease family of proteins. The remaining four — glucose-6-phosphate isomerase (G6PI_HUMAN, [Swiss-Prot:P06744]), aminopeptidase N (AMPN_HUMAN, [Swiss-Prot:P15144]), ATP-dependent RNA helicase DDX51 (DDX51_HUMAN, [Swiss-Prot:Q8N8A6]) and protoheme IX farnesyltransferase (COX10_HUMAN, [Swiss-Prot:Q12887]) — were chosen at random. All results are available by searching for the root protein at http://www.bioinf.org.uk/fosta/.

FOSTA identifies eighteen FEPs for human trypsin-1 (TRY1_HUMAN, [Swiss-Prot:P07477]). Of these, fifteen are clearly trypsin molecules. Some have additional non-functional qualifications (cationic, anionic and alkaline) and demonstrate that FOSTA can make correct FEP assignments despite extraneous information. There are five trypsin FEPs that are not described as trypsin-1 in the second species: TRYB_MANSE, TRYB_DROME, TRYDG_DROER, TRY5_AEDAE and TRYA3_LUCCU. TRYB_DROME and TRYDG_DROER are assigned in favour of several other trypsin proteins in *Drosophila melanogaster* and *Drosophila erecta* respectively. TRYB_MANSE is assigned in favour of TRYA_MANSE and TRYC_MANSE; in *Manduca sexta* (MANSE) it may be that trypsin-B corresponds to human trypsin-1. Without specific information about how and why these proteins were annotated by the respective species annotation communities, it is not clear whether the annotations are misleading, or whether the FOSTA results are incorrect. There are only two trypsin proteins of adequate sequence similarity found in *Aedes aegypti*: TRY5_AEDAE and TRY3_AEDAE. TRY3_AEDAE is equivalent to TRY3_HUMAN and there is no human trypsin-5 protein in UniProtKB/SwissProt version 53.0, so the assignment here appears sensible. In *Lucilia cuprina*, two trypsin proteins are of sufficient sequence similarity: TRYA3_LUCCU, which has been identified as the FEP of TRY1_HUMAN, and TRYA4_LUCCU which has been identified as the FEP of TRY3_HUMAN. This is a difficult assignment to assess, particularly as TRYA3_LUCCU is a fragmented protein. It is worth noting that these five questionable trypsin proteins are derived from insect species:

LUCCU, DROME and DROER are flies, AEDAE is a mosquito and MANSE is a moth. It may be that trypsin genes have duplicated and diverged in insect species.

In addition to the trypsin molecules, FOSTA identifes GRAG_MOUSE, VSP1_BOTJR, VSP1M_TRIST as FEPs because they are described as serine proteases as is TRY1_HUMAN. All mouse proteins explicitly described as trypsin belong to other FOSTA families, with protein prefix matches. There are no trypsin proteins for *Bothrops jararacussu* (BOTJR) or *Trimeresurus stejnegeri* (TRIST), but again it is unclear whether the assignment is correct or not.

FOSTA identifies 25 fully sequenced FEPs and two FEP fragments for aminopeptidase N (AMPN_HUMAN/P15144). There are seven assignments that do not match with respect to protein prefix: AAP1_YEAST, AMP11_ENCCU, AMP1_PLAFQ, AMPM_HELVI, AMPN1_LACLA, APE1_SULSO, APE1_SULTO. AMPN1_LACLA is assigned over the one other *Lactococcus lactis subsp. lactis* candidate (AMPN2_LACLA) as it is of higher sequence identity to AMPN_HUMAN; AMPN2_LACLA is assigned to the FOSTA family of PSA_HUMAN, another aminopeptidase. AMPN1_LACLA matches AMPN_HUMAN with respect to EC number, and contains the same synonym 'Aminopeptidase N'. AMPM_HELVI is the only protein *Heliothis virescens* protein found by the BLAST search, and has a good description field match with AMPN_HUMAN; this appears to be the correct FEP for AMPN_HUMAN in *Heliothis virescens*. APE1_SULSO, APE1_SULTO, AAP1_YEAST, AMP11_ENCCU, AMP1_PLAFQ are the five least reliable assignments, although they are clearly aminopeptidases. Four of the five are flagged as unreliable and so could be removed from FOSTA.

The ATP-dependent RNA helicase DDX51_HUMAN [Swiss-Prot:Q8N8A6] is assigned four full FEPs and four fragmented FEPs by FOSTA. The identification of FEPs for DDX51_HUMAN is a formidable task: DDX51_HUMAN belongs to a large family of 'DEAD box helicases', described by UniProtKB/SwissProt family classifications (http://expasy.org/cgi-bin/get-similar?name=DEAD\%20box\%20helicase\%20family). All four of the fully sequenced proteins (DDX51_DANRE, DDX51_MOUSE, RH1_ARATH and RH1_ORYSJ) belong to the same subfamily as DDX51_HUMAN (the DDX51/DBP6 subfamily). The fragments IF413_TOBAC, DDX6_CAVPO, DDX1_DROVI and IF4A1_RABIT belong to the eIF4A, DDX6/DHH1, DDX1 and eIF4A subfamilies respectively. All proteins assigned to a different subfamily may be misassigned. The

UniProtKB/SwissProt family/domain classifications are manually confirmed, which suggests that in the case of DDX51_HUMAN, the candidate FEPs are so similar that FOSTA finds it difficult to discriminate between them. It should be stressed that a manual analysis of UniProtKB/SwissProt entries for this family is no more effective than FOSTA, and that where FOSTA is incorrect in the DDX51_HUMAN assignments, the proteins are fragments, and flagged as potentially unreliable.

The results for human glucose-6-phosphate isomerase (G6PI_HUMAN, [Swiss-Prot:P06744]) appear very robust: 309 FEPs are identified, of which two are fragments. All of these proteins are glucose-6-phosphate isomerases. Only eighteen of the 309 assignments are made on the basis of sequence (where sequence matching is required to differentiate between G6PI1-4 or G6PIA-B proteins) and 287 (92.88% of these are protein prefix matches). As already discussed, without explanation of how these proteins were named, it is not clear whether FOSTA is generating the correct pairs, or whether the sequence matching is misleading.

FOSTA identifies 34 FEPs for human protoheme IX farnesyltransferase (COX10_HUMAN, [Swiss-Prot:Q12887]), all of which are fully sequenced proteins. These results appear very reliable, with only one FEP chosen from all candidates on the basis of sequence identity, where COXX_BACSU is chosen over CTAO_BACSU. Given that these two proteins are annotated identically in UniProtKB/SwissProt, it is reasonable to resort to sequence similarity to discriminate between them. The results are particularly encouraging given that, unlike most G6PI_HUMAN FEPs, protoheme IX farnesyltransferases have different UniProtKB/SwissProt protein prefixes in different species.

Random samples used to compare FOSTA with Inparanoid

For the random samples used to compare FOSTA with Inparanoid, see Tables 1 and 2.

PIRSF/Hulsen benchmarking: additional statistics

PPV and MCC were included in the main text. Here we include specificity and sensitivity for both datasets (Table 3 for PIRSF and Table 4 for completeness.

Table 1: A random sample of ten overlooked IP (Inparanoid pairs)
These are overlooked in FOSTA (i.e., FOSTA assigns a different FEP from that species).

**Human**	**Inparanoid**	**FOSTA**
PLCH_HUMAN	PLCHB_DANRE	PLCHA_DANRE
MOXD1_HUMAN	MOX11_DROME	PHM_DROME
EPN4_HUMAN	ENT3_YEAST	ENT4_YEAST
WDR59_HUMAN	YD128_YEAST	YBK4_YEAST
CP2B6_HUMAN	CP2BB_CANFA	CP2CL_CANFA
IF4A3_HUMAN	FAL1_YEAST	IF4A_YEAST
O5AP2_HUMAN	O1020_MOUSE	O1086_MOUSE
DDX6_HUMAN	DHH1_YEAST	DBP6_YEAST
MCM4_HUMAN	CDC54_YEAST	CDC47_YEAST
OR5J2_HUMAN	O1052_MOUSE	O1094_MOUSE

Table 2: A random sample of 28 rejected IP (Inparanoid pairs)
These are rejected in FOSTA (i.e., FOSTA does not assign any FEP from that species).

**Human**	**Inparanoid**
TIM_HUMAN	TOF1_YEAST
CC45L_HUMAN	CDC45_YEAST
SURF1_HUMAN	SHY1_YEAST
TEX10_HUMAN	IPI1_YEAST
FA2H_HUMAN	SCS7_YEAST
IPO9_HUMAN	IMB5_YEAST
ISK5_HUMAN	IOV7_CHICK
LETM1_HUMAN	A60DA_DROME
FRK_HUMAN	SRC42_DROME
NVL_HUMAN	RIX7_YEAST
MMS19_HUMAN	MET18_YEAST
DYR1A_HUMAN	MNB_DROME
ATBP3_HUMAN	NCS6_YEAST
DCR1A_HUMAN	PSO2_YEAST
PDXK_HUMAN	BUD16_YEAST
PLAP_HUMAN	DOA1_YEAST
JAZF1_HUMAN	SFP1_YEAST
EXTL3_HUMAN	EXT3_DROME
ZUBR1_HUMAN	POE_DROME
IPO11_HUMAN	KA120_YEAST
RBBP6_HUMAN	MPE1_YEAST
TRIPC_HUMAN	UFD4_YEAST
PAP1L_HUMAN	EPAB_XENTR
PINX1_HUMAN	YG5W_YEAST
CFDP1_HUMAN	SWC5_YEAST
FGF17_HUMAN	FG17B_DANRE
XPOT_HUMAN	LOS1_YEAST
TM11A_HUMAN	DESC4_RAT

Table 3: Benchmarking FOSTA against the PIRSF dataset, extended statistics

Set	Fams	Pairs	Basic statistics				Evaluation statistics			
			TP	FP	TN	FN	spec	sens	PPV	MCC
A	122	2127	1744	2	3717	383	99.95	81.99	99.89	0.86
B	1095	18865	12967	23	34656	5898	99.93	68.74	99.82	0.77
C	474	11221	9146	62	11819	2075	99.48	81.51	99.33	0.83
D	339	5287	3674	16	4938	1613	99.68	69.49	99.57	0.72
N	1691	32213	23857	87	50192	8356	99.83	74.06	99.64	0.79
$*$	2020	37500	27531	103	55130	9969	99.81	73.42	99.63	0.79

Set: the identifier for each curation set [A='Full/Desc.', B='Full', C='Preliminary', D='None', N=aNnotated (A+B+C), $*$=All (N+D)]; **Curation string**: the string that defines the curation set; **Families**: the number of discrete protein families in the curation set; **Pairings**: the number of discrete pairings across all families to be tested in FOSTA; **Basic statistics**: the basic counts of true positives (TP), false positives (FP), true negatives (TN), false negatives (FN); **Evaluation statistics**: the **PPV** (positive predictive value, $TP/(TP + FP)$), and the **MCC** (Matthews Correlation Coefficient), all rounded to 2dp

Table 4: Benchmarking FOSTA against the refined Hulsen *et al* dataset, extended statistics

Family	Refined	(TO)	Basic statistics				Evaluation statistics			
			TP	FP	TN	FN	spec	sens	PPV	MCC
HBB	2	(9)	2	0	17	0	100.00	100.00	100.00	1.00
HOX	30	(41)	30	0	3853	0	100.00	100.00	100.00	1.00
SMm	12	(17)	12	0	22	0	100.00	100.00	100.00	1.00
SMc	6	(6)	6	0	5	0	100.00	100.00	100.00	1.00
NR	4	(29)	1	1	327	3	99.70	25.00	50.00	0.35
All	54	(102)	51	1	4224	3	99.98	94.44	98.08	0.96

Protein family: the protein family being examined; **TO pairings**: the number of TO pairs in the Hulsen dataset (including many-to-many orthologous pairings and non-UniProtKB/SwissProt proteins); **Refined pairings**: the number of one-to-one TO pairings tested after refinement of Hulsen TO dataset; **Basic statistics**: the basic counts of true positives (TP), false positives (FP), true negatives (TN), false negatives (FN); **Evaluation statistics**: the **PPV** (positive predictive value, $TP/(TP + FP)$), and the **MCC** (Matthews Correlation Coefficient), all rounded to 2dp)
